# Supplementary material for: Radially distributed charging time constants at an electrode-solution interface
Source: Nat Commun. 2024 Jul 4;15:5633. doi: 10.1038/s41467-024-50028-2 (PMC11224254; doi:10.1038/s41467-024-50028-2)
Supplement: Supplementary file 1 — Supplementary information [file 41467_2024_50028_MOESM1_ESM.pdf]

Supplementary Information for

Radially distributed charging time constants at an electrode-solution interface

Ben Niu[1†], Ruo-Chen Xie[1†], Bin Ren,[2,3] Yi-Tao Long[1], and Wei Wang[1*]

[1]State Key Laboratory of Analytical Chemistry for Life Science, School of Chemistry and Chemical Engineering, Chemistry and Biomedicine Innovation Center (ChemBIC), Nanjing University, Nanjing, 210023, China

[2]State Key Laboratory of Physical Chemistry of Solid Surfaces, Collaborative Innovation Center of Chemistry for Energy Materials (i-ChEM), Department of Chemistry, College of Chemistry and Chemical Engineering, Xiamen University, Xiamen 361005, China.

[3]Innovation Laboratory for Sciences and Technologies of Energy Materials of Fujian Province (IKKEM), Xiamen 361005, China.

* Corresponding author. E-mail: wei.wang@nju.edu.cn

Table of contents

Supplementary Notes

1. Electrochemical reflective spectroscopy of the gold macroelectrode
2. Removal of short-term noises by introducing a silicon optical reference
3. Surface morphology characterization of the gold disk electrode using tilted SEM
4. Radial distribution of reduction peak potentials at various scan rates and electrolyte concentrations
5. Peak potentials difference across a Prussian blue film electrode surface
6. Finite element simulation of proton diffusion during the gold reduction
7. Solution resistance distribution under strong convection conditions
8. Charge estimation from the electrical cyclic voltammograms
9. Time constant distribution of the charging process
10. Negligible effects of uneven illumination and focal plane on the optical measurements
11. Electrochemical impedance imaging of the gold electrode
12. R_s distribution on a GC electrode
13. Theoretical modelling based on COMSOL
14. R_s distribution on the electrode passivated with an insulating boundary
15. R_s distributions on the retracted electrode

Supplementary References

Supplementary Notes

1. Electrochemical reflective spectroscopy of the gold macroelectrode

In order to reveal the source of optical signals, we collects the transient reflective spectroscopy of the gold electrode during the redox process, using a halogen lamp as the light source. A grating spectrometer (Acton Spectra Pro SP-2300, Princeton Instruments) with slit and EMCCD (ProEM+, 1024B eXcelon, Princeton Instruments) is used to capture the reflective spectra at different potentials (Supplementary Fig. 1a). At 0.05 V, we define the reflectivity at all wavelengths as 100%. By comparing the reflectivity (I / I_0) at the oxidation potential of 1.5 V, we observe a distinct maximum of the changes in reflectivity at ~520 nm (Supplementary Fig. 1b), a characteristic that is probably associated with absorption gold or scattering of surface nanogold [1,2]. This finding indicates that the changes in the optical reflectivity predominantly result from the variations in the dielectric constant of gold electrode itself [3]. Consequently, we choose 530 nm LED as the light source for the optimal sensitivity of the optical measurements throughout the work.

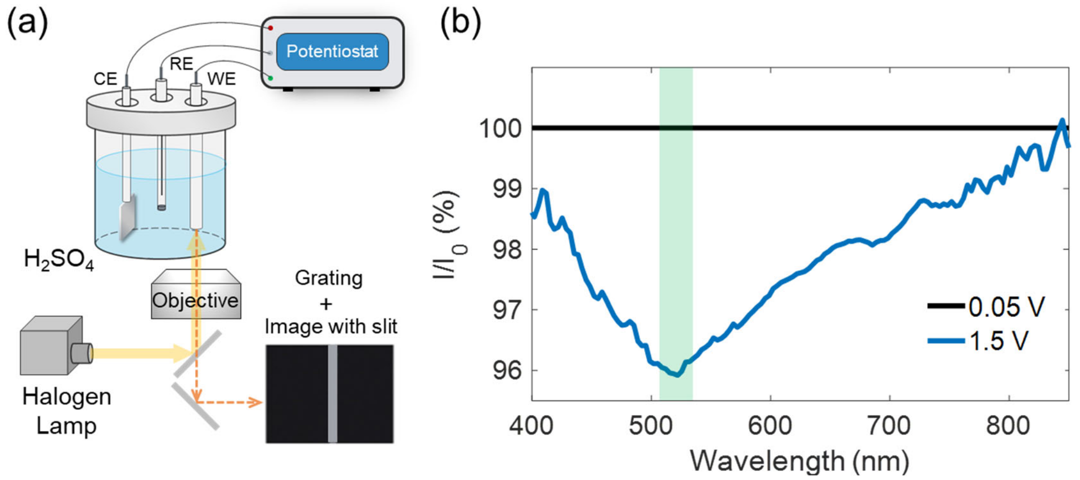

Supplementary Fig. 1. (a) Schematics of the electrochemical reflective microscopy for spectroscopic measurements. (b) The normalized reflective spectra of the gold electrode at 0.05 V and 1.5 V during the cyclic voltammetry process. The green area represents the wavelength bandwidth of the 530 nm LED.

2. Removal of short-term noises by introducing a silicon optical reference

Inspired by our previous work [4], a first-order derivative has to be performed to the optical reflectivity curves to obtain the optical current. Since the change in optical

intensity corresponds to the change of electrochemical charge, differentiating the optical intensity over time results in the optical current (corresponding to charge transfer rate). However, the first-order derivative is known to amplify the short-term noise caused by the light source.

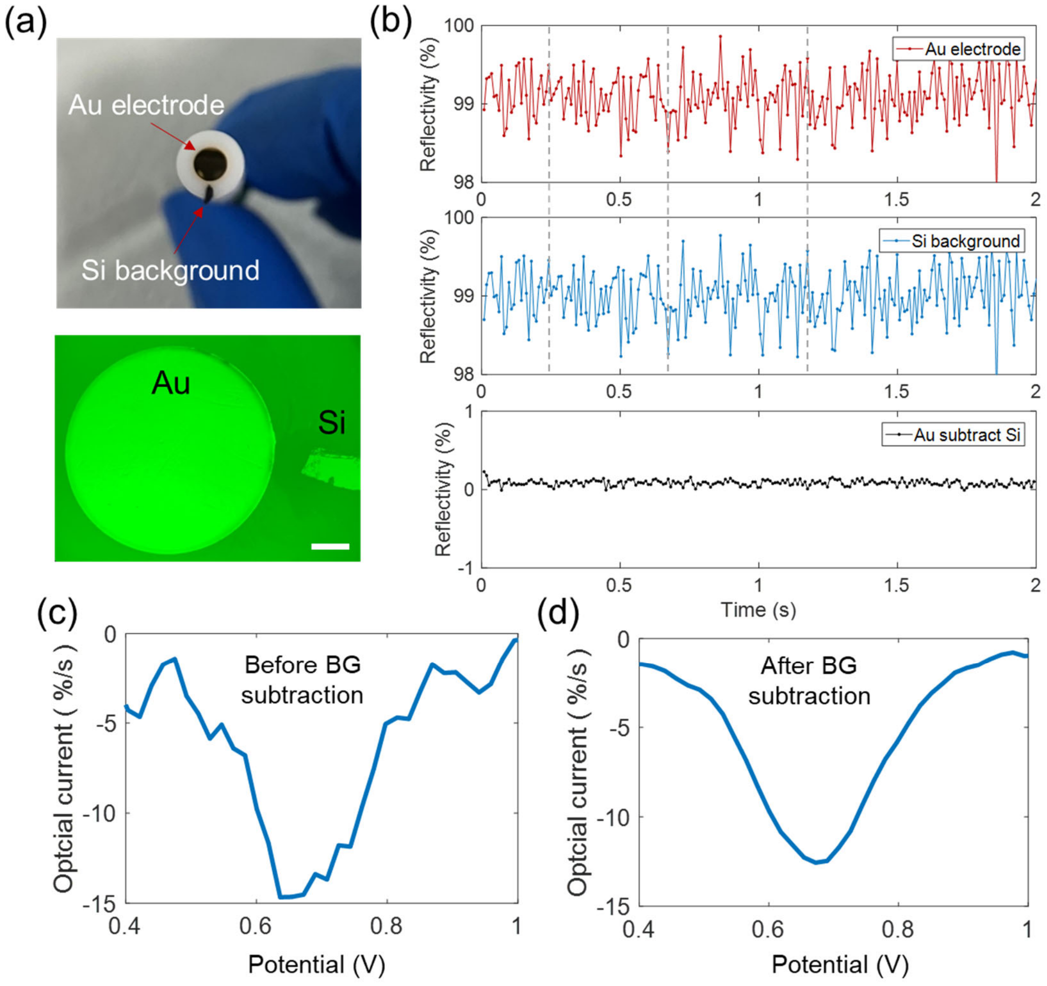

Supplementary Fig. 2. (a) Photograph of a gold disc electrode with adjacently immobilized silicon wafer (top panel) and the corresponding optical image under the green LED illumination (bottom plane), scale bar 0.5 mm. (b) Reflectivity of the Au electrode (top panel) and the Si background (middle panel) as a function of time. The bottom panel is the subtracted reflectivity curve. (c) and (d) are the corresponding optical CV curves obtained before and after the Si background subtraction, scan rate: 1V/s.

To eliminate the short-term noise in the measured optical signals, we introduced an optical reference of a small piece of silicon wafer. The piece of solid was fixed on the resin sheath surface of the gold disk electrode (Supplementary Fig. 2a) and its surface was place parallel and adjacent to the gold surface ensuring a similar imaging to the conditions for the gold surface and preventing any disruption to mass transport.

Consequently, a highly synchronized intensity fluctuation was observed between the gold surface and the reference silicon surface (Supplementary Fig. 2b). By subtracting the optical reflectivity curve of the gold from that of the silicon, a significant removal in short-term noise was achieved, corresponding to an impressive 8.5-fold increase in the signal-to-noise ratio. This enhancement and the following Gaussian fitting facilitated the precise extraction of peak potentials at every electrode location (Supplementary Fig. 2c-d).

By employing background subtraction and Gaussian fitting strategies, we have achieved a large signal-to-noise (~100) ratio across the entire distribution, despite the reflectivity exhibiting fluctuations of less than 4% in value. The reproducibility of the same experiments is high, altogether leading to a negligible standard deviation of around 1 mV at most.

3. Surface morphology characterization of the gold disk electrode using tilted SEM

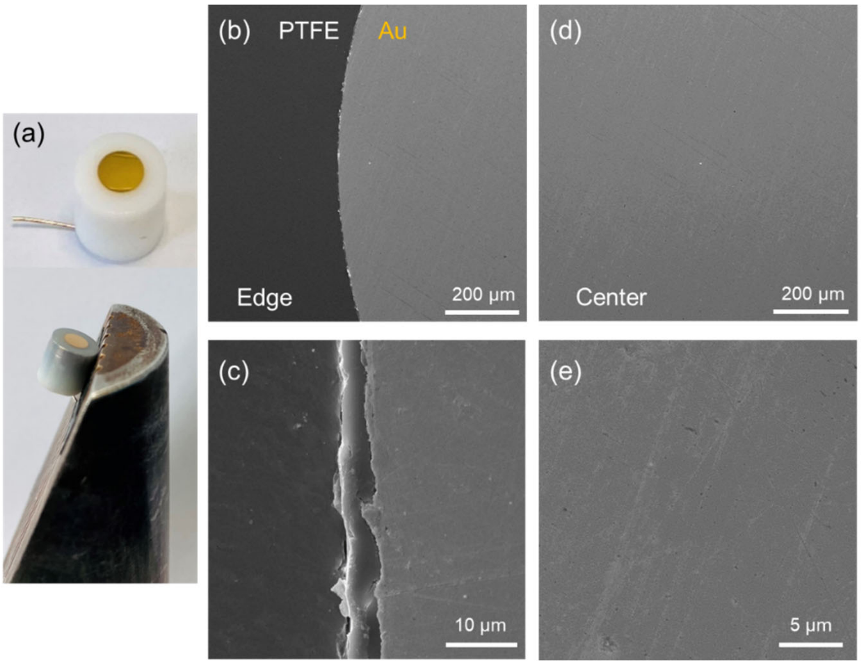

Supplementary Fig. 3. (a) Top view of a PTFE-encapsulated gold electrode and side view of the sputtered gold electrode loaded on to the SEM sampler at a tilted angle of ca. 15°. SEM images of the electrode edge (b-c) and central (d-e) regions.

The morphology of the gold disk electrode has been characterized at an inclined angle using scanning electron microscopy (Supplementary Fig. 3a). The SEM results reveal a highly uniform surface morphology of the electrode without any noticeable surface curvature at both the electrode periphery (Supplementary Fig. 3b&c) and the

middle areas (Supplementary Fig. 3d&e). There are only some randomly distributed scratches at the micrometer scale and a few visible defects, likely arising from the manual polishing.

4. Radial distribution of reduction peak potentials at various scan rates and electrolyte concentrations

The column dimension of Supplementary Fig. 4 showed the relative peak potentials (to the value of central positions) of the gold reduction process radially across the entire electrode surface as a function of scan rate. As the scanning rates increased from 0.1 V/s to 1/V s, the observed peak potential discrepancy between the central and periphery regions increased from 2.2 mV (0.1 V/s), 13 mV (0.5 V/s) to 24 mV (1 V/s) respectively, displaying nearly liner variations. However, although the discrepancy was more prominent at fast scans, the differences in time required to reach the peak current remains constant at ~25 milliseconds (peak potential difference divided by scan rate), regardless of scan rate.

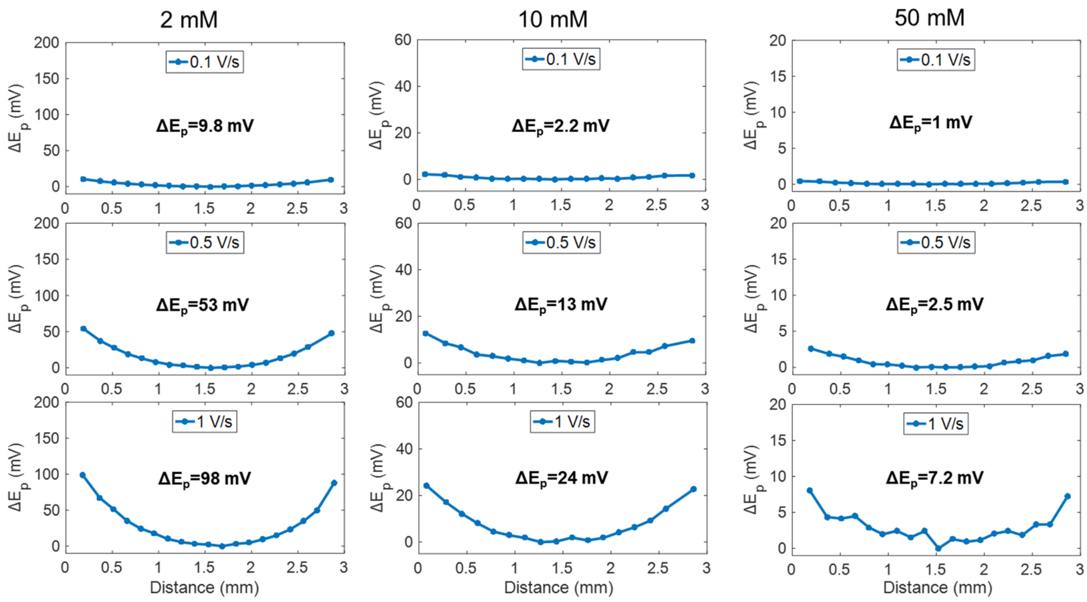

Supplementary Fig. 4. Radial distribution curves of peak potential difference of the same Au electrode at scanning rates of 0.1 V/s (top panel), 0.5 V/s (middle panel), 1V/s (bottom panel) and sulfuric acid concentrations of 2 mM (left panel), 10 mM (central panel) and 50 mM (right panel).

The spatial variations in peak potentials are investigated also at different concentrations of sulfuric acid (2 mM, 10 mM, and 50 mM). The row dimension of Supplementary Fig. 4 showed the consistent presence of a hump-like radial distribution,

irrespective of the acid concentrations. The spatial differences are the most pronounced with the lowest electrolyte concentration: in a 2 mM H_2SO_4 solution, the time differences could reach up to 98 ms. Reversely, a high concentration of 50 mM made the spatial differences almost not visible but considering the overall knowledge, we believe this was merely buried by the measurement noise rather than physical elimination.

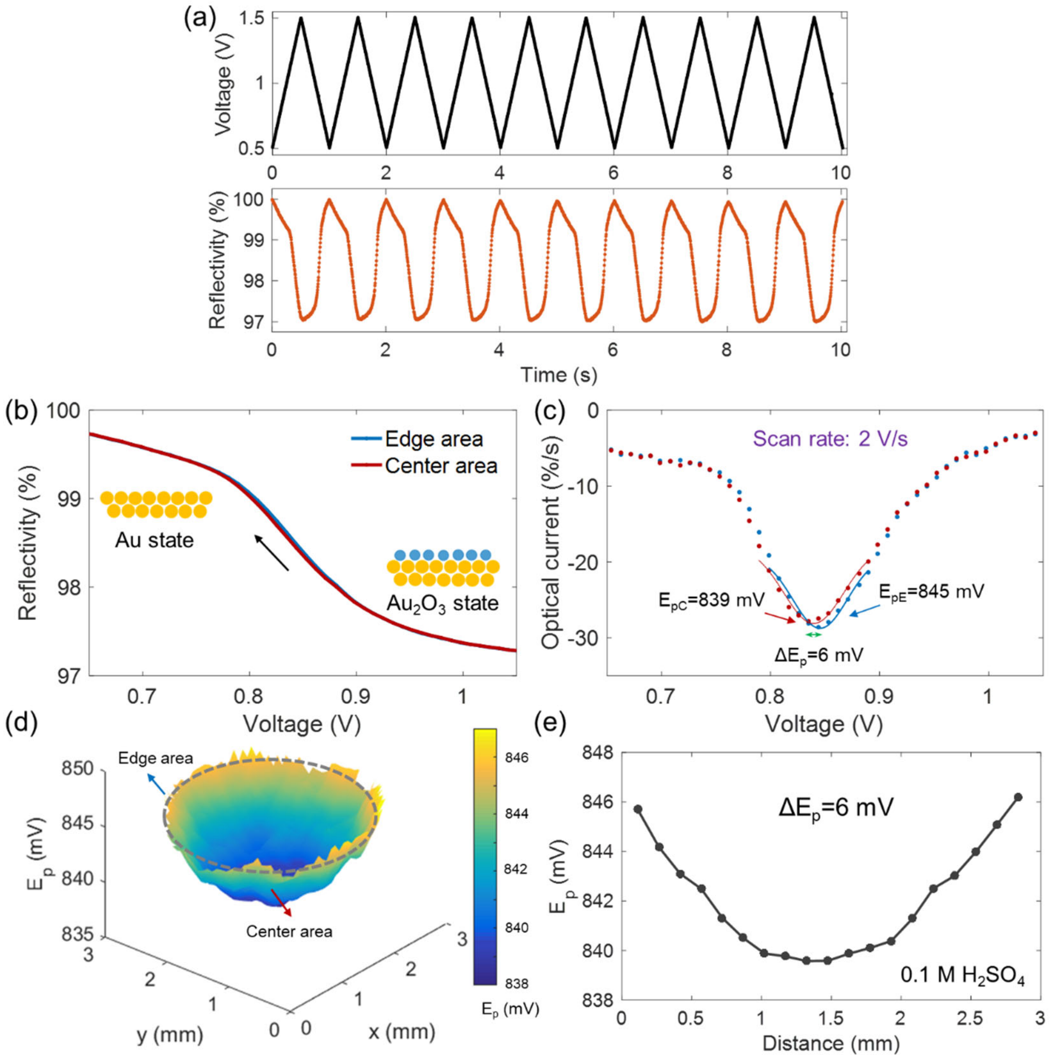

Supplementary Fig. 5. (a) Consecutive triangular wave potential applied between 0.5 and 1.5 V and the corresponding optical reflectivity curve of the selected 8 consecutive cycles. (b) Potential dependent optical reflectivity measured for the edge and central regions respectively. Scan rate: 2 V/s, solution: 0.1 M sulfuric acid. (c) Reductive optical voltammetric peaks of the peripheral and central electrode areas. The line is the fitted results, and the dots are experimental data. (d) Pixel-level peak potential distribution over the whole electrode surface and (e) the representative radial peak potential distribution.

Given that conventional electrochemical systems typically operated with solution concentrations exceeding 0.1 M, it is important to investigate further at these conditions. However, as the concentration increases the spatial differences decreases drastically. Considering that the establishment of the interfacial potential would occur much faster at the very high electrolyte concentration, one can use a higher scan rate for faster optical measurements. In addition, minimizing experimental noise is also crucial for attaining a clear reduction peak potential variation at the concentrated electrolyte.

To do so, we adopt a strategy of averaging multiple signal cycles to enhance the signal-to-noise ratio. By applying a periodic triangular wave potential and capturing 100 cycles at a steady rate of 500 frames per second (Supplementary Fig. 5a), each cycle was then superimposed with precise alignment, resulting in a reflectivity profile with a high signal-to-noise ratio (~500) as shown in Supplementary Fig. 5b. In the case of 0.1 M sulfuric acid, the corresponding reduction peak potential results reveal a small but clear (approximately 6 mV) peak potential differences between the edge and central regions (Supplementary Fig. 5c). At the same time, we also map peak potentials distribution and radial distribution curve (Supplementary Fig. 5d&e). Employing this averaging strategy also yielded similar radial distribution curves even in a higher 0.5 M sulfuric acid solution setting (Supplementary Fig. 6). After all, the spatial discrepancy was dominated by the solution side.

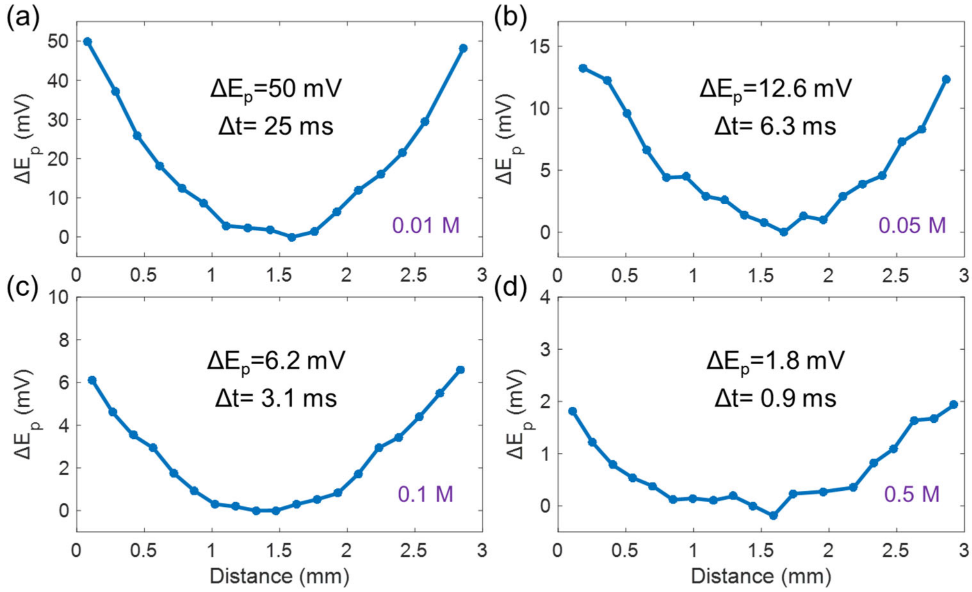

Supplementary Fig. 6. Radial distribution curves of peak potential difference in (a) 0.01 M, (b) 0.05 M, (c) 0.1 M, and (d) 0.5 M sulfuric acid solutions, scan rate: 2 $V \cdot s^{-1}$.

Combined with the overall conclusions of our study, we delved into the impact of solution resistance on the Faraday electron transfer reaction. The primary driver

behind the spatial variance in peak potential was identified as the ohmic resistance (IR drop) within the solution. At the scan rate of 1 V·s^{-1}, the average reduction peak current in electric CV was approximately -0.19 mA, with an average solution resistance of 245 Ω. Accordingly, the potential lost attributed to the solution's IR drop was estimated at around 46.5 mV. Considering a 60% disparity in solution resistance in Fig. 5d between the edge and central regions, the resulting difference in potential amounted to be approximately 28 mV, corresponding to a time differential of 28 ms, in great agreement with experimental observations.

The IR drop in the central region led to a more negative potential being applied to counteract this impedance, even though the reaction can occur at the same potential. In the case of the gold reduction, an additional potential of -24 mV must to be supplied to achieve the intrinsic potential required to initiate the reaction, leading to a notably reduced value for the reduction peak potential. At the same time, the relationship between scan rate and current intensity ($i \propto v$) indicated a direct rise in the magnitude of the IR drop and subsequent disparity in peak potentials, which is consistent with the results in Supplementary Fig. 5. Furthermore, the solution resistance R_s emerged as a crucial contributor in IR drop, introducing a time delay that caused regions with higher resistance to achieve the real reaction potential at a delayed pace. The presence of this IR drop at the electrode surface prompts a critical reassessment of whether parameters derived from electrocatalysis and electroplating experiments authentically reflect the material's intrinsic characteristics.

5. Peak potentials difference across a Prussian blue film electrode surface

Apart from the reduction of gold oxide, the electrochemical characterization of another redox active, optically responsive material Prussian blue (PB, $KFe^{III}Fe^{II}(CN)_6$, an electrochromic substance) film was conducted with both focuses on the oxidation and reduction processes. Experimentally, Prussian blue films were prepared by electrodeposition on gold electrodes in a solution containing 1.0 mM $FeCl_3 \cdot 6H_2O$, 1.0 mM $K_3Fe(CN)_6$, 0.1 M KCl and 25 mM HCl (AR grade, Sinopharm Chemical Reagent Co,. Ltd). Cyclic voltammetry between 0 and 0.8 V was imposed on the electrode for 2 cycles at a scan rate of 50 mV/s. The obtained film was subsequently washed with deionized water and dried in vacuum at 30°C for 3 hours [5].

The following electrochemical measurements were carried out in a 10 mM KNO_3 solution. Note that the optical system is similar to that used in the Au redox experiments, except the use of a 730 nm LED at this time (M730L4-C1, Thorlabs) due to the

absorptive spectral peak of PB at approximately 700 nm [6]. A clear dependence of the reflectivity with the electrode potential was observed (Supplementary Fig. 7a), with the absorbance of the reduced state (Prussian white, PW, $K_2Fe^{II}Fe^{II}(CN)_6$) being lower than that of the oxidized state (PB). The first-order derivatives of the measured reflectivity as a function of the applied potential for different regions were then obtained as optical CV. Notably, the edge region still displayed an earlier reaction compared to the central region, with a peak potential difference of approximately 40 mV, regardless of reduction or oxidation process (Supplementary Fig. 7b).

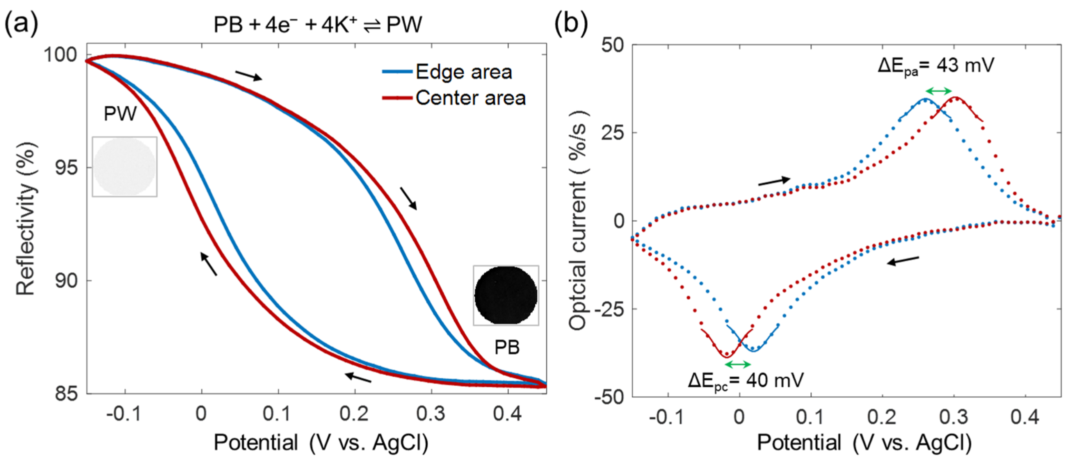

Supplementary Fig. 7. (a) Potential dependent optical reflectivity measured for the edge and center areas respectively. (b) Optical voltammetry of the edge and center areas respectively. The line is the fitted results, and the dots are experimental data.

6. Finite element simulation of proton diffusion during the gold reduction

We conduct the FE simulation of the 1.5 mm-radius electrode charging processes at different timescales from 100 ms to 5 s. The results show that the radial diffusion can be significant at the scale of ca. 500 μm but in this case the diffusion layer has evolved for 5 seconds, greatly longer than our timescale of interest (10 ms). Importantly at this stage, the diffusion layer above the central region of the electrode does not appear any significant extent of heterogeneity across the electrode surface. The region with significant radial diffusion only shrinks to a lengthscale of around 200 microns at 1 second and further down to 50 microns at 100 ms. (Supplementary Fig. 8)

It is thus emphasized that the key difference between our observed phenomenon and the well-known radial diffusion at electrode edges is, at least in part, the continuity of the spatial differences across the whole electrode surface. Consequently, with these simulation results, we further confirm the exclusion of a significant contribution from

radial diffusion.

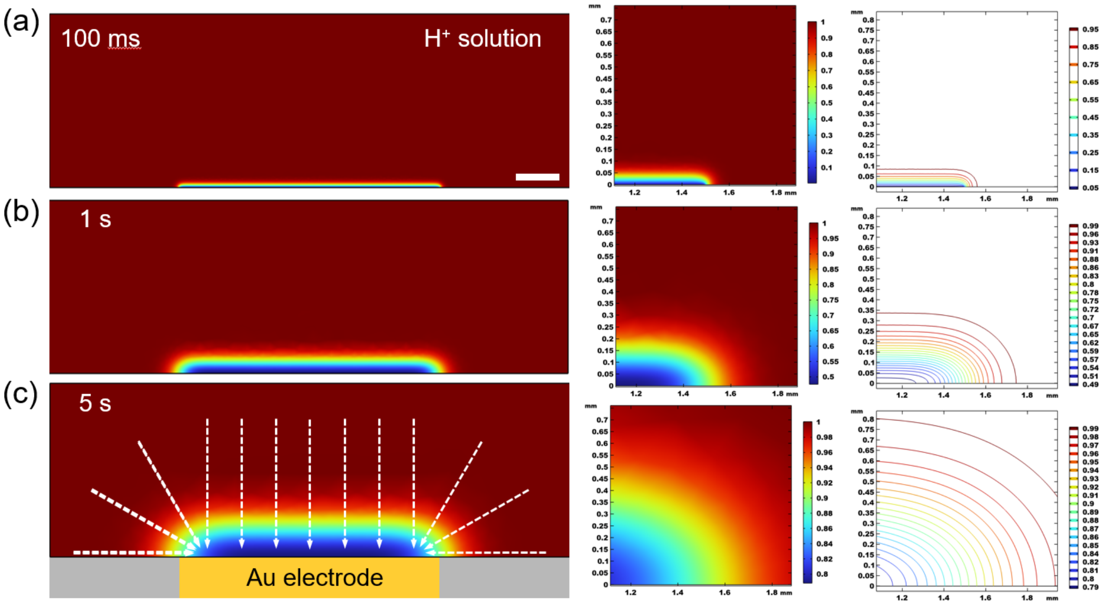

Supplementary Fig. 8. Side view of the distribution of proton profiles above the electrode surface at different timescales from (a) 100 ms, (b) 1 s and (c) 5 s, scale bar 0.5 mm.

7. Solution resistance distribution under strong convection conditions

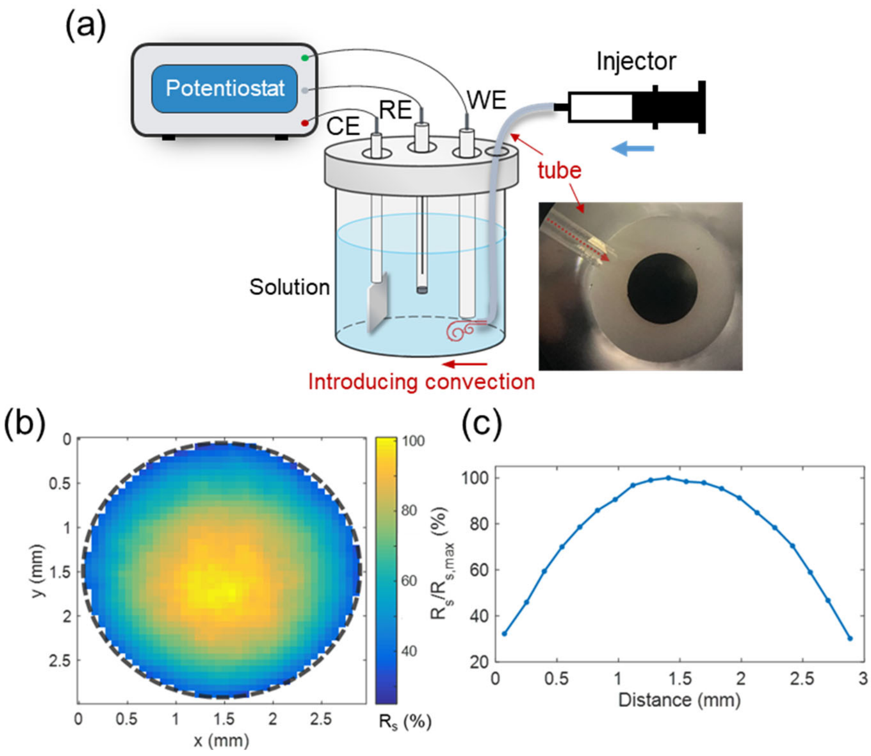

Supplementary Fig. 9. (a) Experimental setup of hydrodynamic experiments and corresponding (b) mapping and (c) distribution of the measured solution resistance.

To introduce hydrodynamics into the solution, we positioned an injector near the electrode surface to induce strong convection in the near-electrode space (Supplementary Fig. 9a). Even so, we still observed the hump-like optical impedance and thus the solution resistance distribution. This experiment indicated that what the solution was doing (especially diffusion) did not affect solution resistance, and the latter was mainly determined by the electric field distribution between the WE and CE. These findings provide additional evidence supporting the relationship between solution resistance and electric field intensity and excluding the contribution from radial diffusion.

8. Charge estimation from the electrical cyclic voltammograms

Given the atomic radius (144 pm) of gold in the close-packed solid phase, the surface area of the well-polished electrode (7.07 mm^2) and a three-electron-transfer reduction of gold to gold oxide, the estimated charge expected from a monolayer reaction is calculated to be 5.1×10^{-5} C. By contrast, a columbic estimate of the surface oxide formation from the electrical CV gives a value of 5.5×10^{-5} C. The consistency confirms the occurrence of a monolayer reaction. The roughness factor (RF), calculated by dividing the real surface area by the geometric surface area [7], is found to be approximately 1.1.

9. Time constant distribution of the charging process

Obtaining curve with a high SNR is not straightforward in the fast charging process (over ~ 20 ms) and, as we have managed, requires averaging numbers of repeats of the potential step, due to the minimal reflectivity variations of only 0.2%. To this end, a periodic square wave was employed, recording 500 cycles at constant speeds of 500 frames per second. Each cycle was then superimposed with precise alignment, resulting in a finally much clearer representation of the charging process as depicted in Supplementary Fig. 10.

Next, the reaction kinetics of Au electrode in charging process is described by the following exponential formula:

$$Q = a \cdot (1 - exp\left(-\frac{t}{\tau}\right)) \quad (1)$$

where *Q* is the transferred charge during the electrode process, *a* is a pre-exponential constant related to the initial state of the electron density of the electrode, *t* is the charging time and τ is the time constant. The equation is derived from the current

integration of an RC circuit charging mechanism [8]. Supplementary Fig. 8b illustrated he fitted curves for both the edge and central regions, showing discrepancies between the two areas. As described in the main text, the rise in the optical reflectivity occurred earlier in the edge area. Consequently, the spatial distribution of the charging rate can be depicted in terms of the time constant (τ) in Fig. 3c. The similar hump-like curve of time constant was observed (Supplementary Fig. 10c).

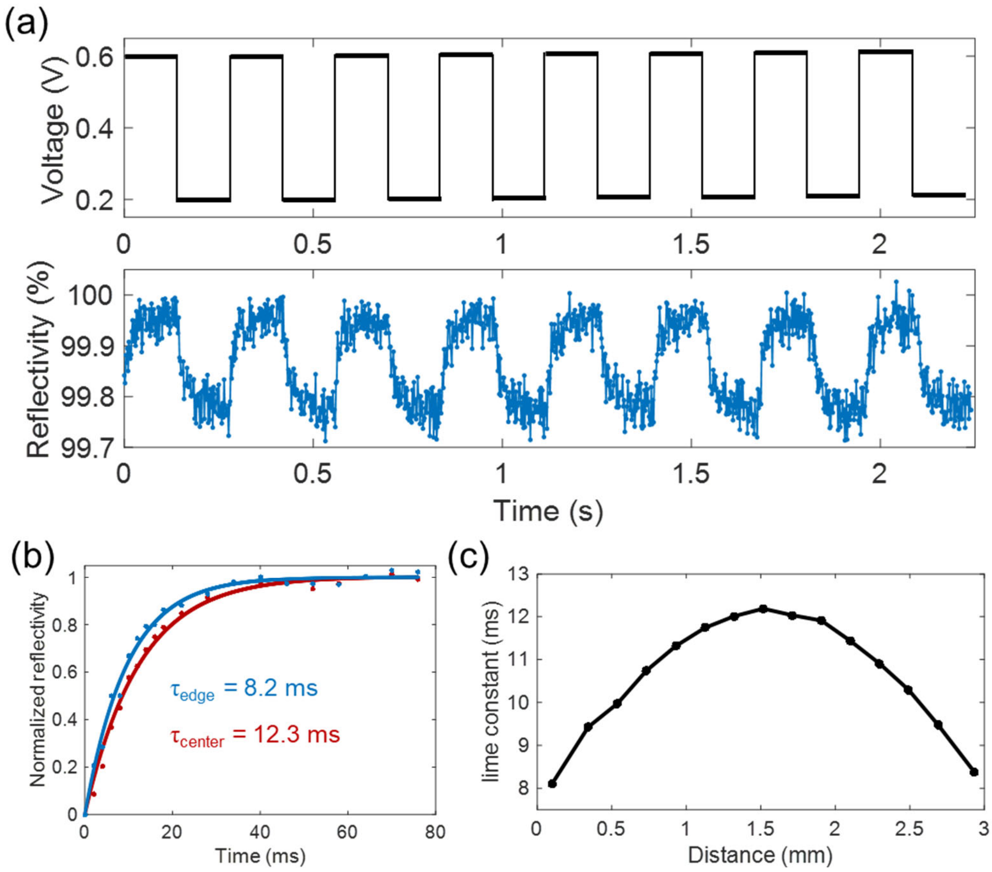

Supplementary Fig. 10. (a) The applied potential steps between 0.6 and 0.2 V and the corresponding reflectivity curve of the selected 8 consecutive cycles. (b) The fitted results of both edge and center area. The line is the fitted results, and the dots are experimental data. (c) The representative radial time constant distribution curve.

At the same time, we again check the correlation between the current, charge, and optical reflectivity curve in response to a potential step ranging from 0.6 V to 0.2 V. Notably, the comparison of the charge curve with the reflectivity curve confirm remarkably strong correlation (Supplementary Fig. 11).

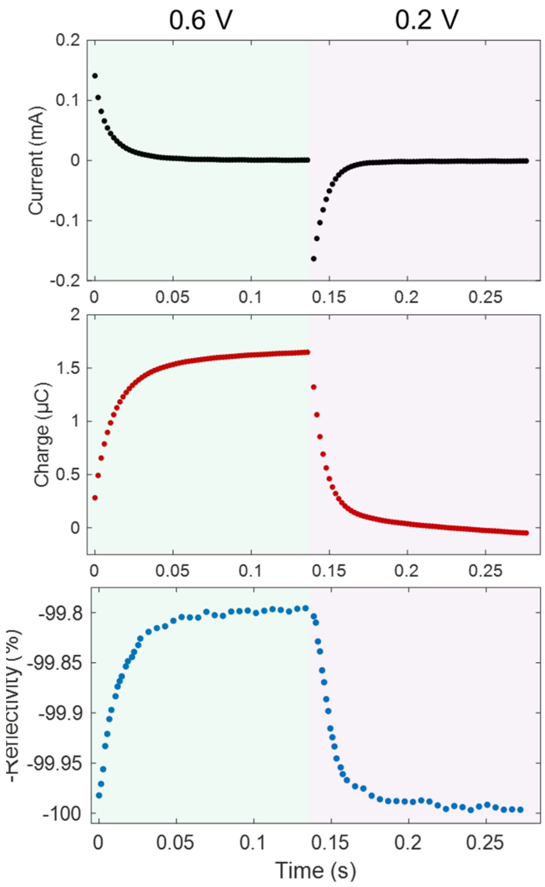

Supplementary Fig. 11. Current curve (top panel), charge curve (middle panel) and optical reflectivity curve (bottom panel) during potential step for the whole gold electrode.

10. Negligible effects of uneven illumination and focal plane on the optical measurements

Technical concerns of uneven illumination and focal plane are discussed. We deliberately make the illumination greatly inhomogeneous, that is, the intensity drastically varying across (only) one radial direction. We then perform the same experiment, and the qualitatively same pattern is obtained again (Supplementary Figure 12d-f). Although the latter appears noisier, this indicates that the pattern discovered is not affected by the uneven illumination.

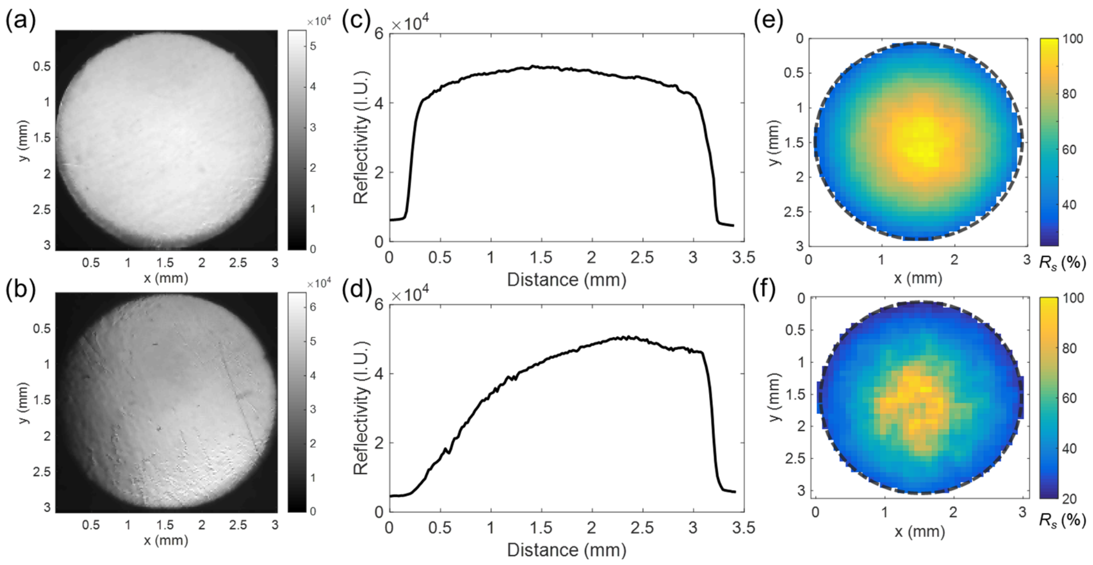

Supplementary Fig. 12. Images of the electrode surface with (a) the relatively even and (b) rather uneven illumination. The corresponding (c, d) initial reflectivity distribution across the electrode surface and (e, f) the experimentally measured solution resistance distributions.

To resolve the effect of focal plane on the optical measurements, we deliberately move the focal plane very far (200 microns) away from the geometrical plane of the gold electrode (Supplementary Fig. 13.). As a result, the reported spatial differences are still present. It is thus reasonable to infer that the potential-modulated double layer that can theoretically change the focal position during voltammetry are not a significant aspect to affect the optical measurements.

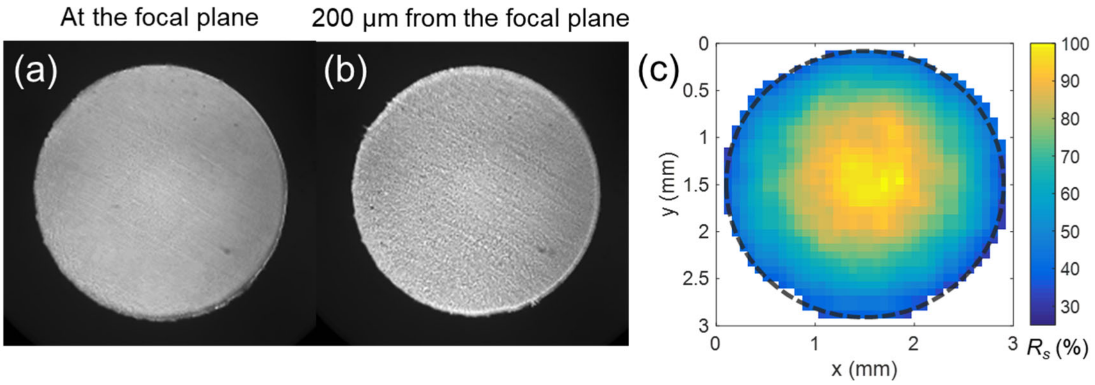

Supplementary Fig. 13. Reflectivity of the gold (a) at and (b) 200 microns away from the focal plane. (c) Solution resistance mapping of the latter situation.

11. Electrochemical impedance imaging of the gold electrode

Electrically, the EIS of the gold electrode is first measured and analyzed using Randles equivalent circuit (Supplementary Fig. 14). The solution resistance R_s was

determined to be 1.95 kΩ, the double-layer capacitance C_{dl} was 2.08 μF. Considering an electrode surface area of ~7.07 mm^2, the surface capacitance was calculated to be 29.4 $\mu F/cm^2$, agreed with the theoretical value [9].

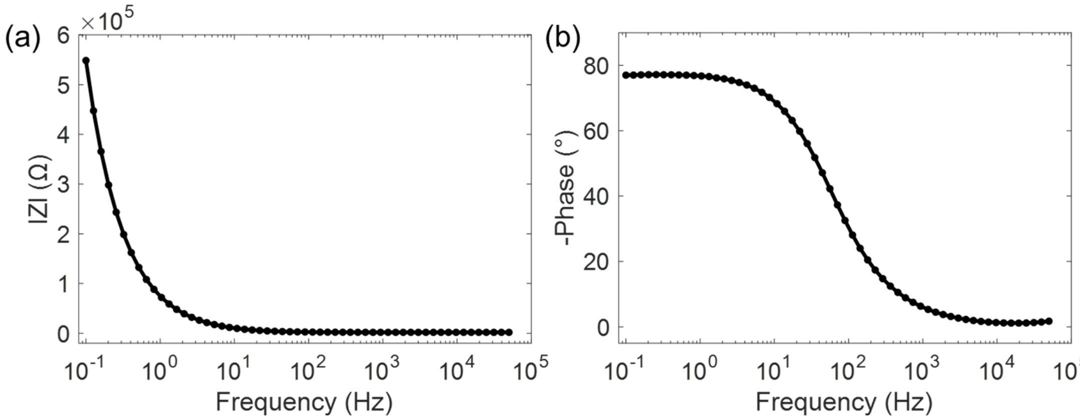

Supplementary Fig. 14. Bode plots of electrical impedance modulus (a) and phase (b) of the overall electrochemical cell.

Next, the optical electrochemical impedance spectroscopy (oEIS) measurement uses a sinusoidal potential modulation with an amplitude of 100 mV (offset, 400 mV) applied over a frequency range of 0.1 Hz to 200 Hz (Supplementary Fig. 15a). In Supplementary Fig. 15b, the optical amplitude was found to be approximately 0.05% at 0.1 Hz.

The optical amplitude at each frequency was then extracted using the Fourier transform, showcasing a robust signal-to-noise ratio even at high frequencies (Supplementary Fig. 15c-d). Subsequently, we extracted the spatial distribution of optical amplitudes across the surface of electrode at 0.1 Hz (Supplementary Fig. 16a) and 100 Hz (Supplementary Fig. 16b). The results revealed a uniform optical amplitude profile at low frequency (0.1 Hz) and a pronounced spatial heterogeneity at high frequency (100 Hz), with the edge region exhibiting significantly higher optical amplitude compared to the central region.

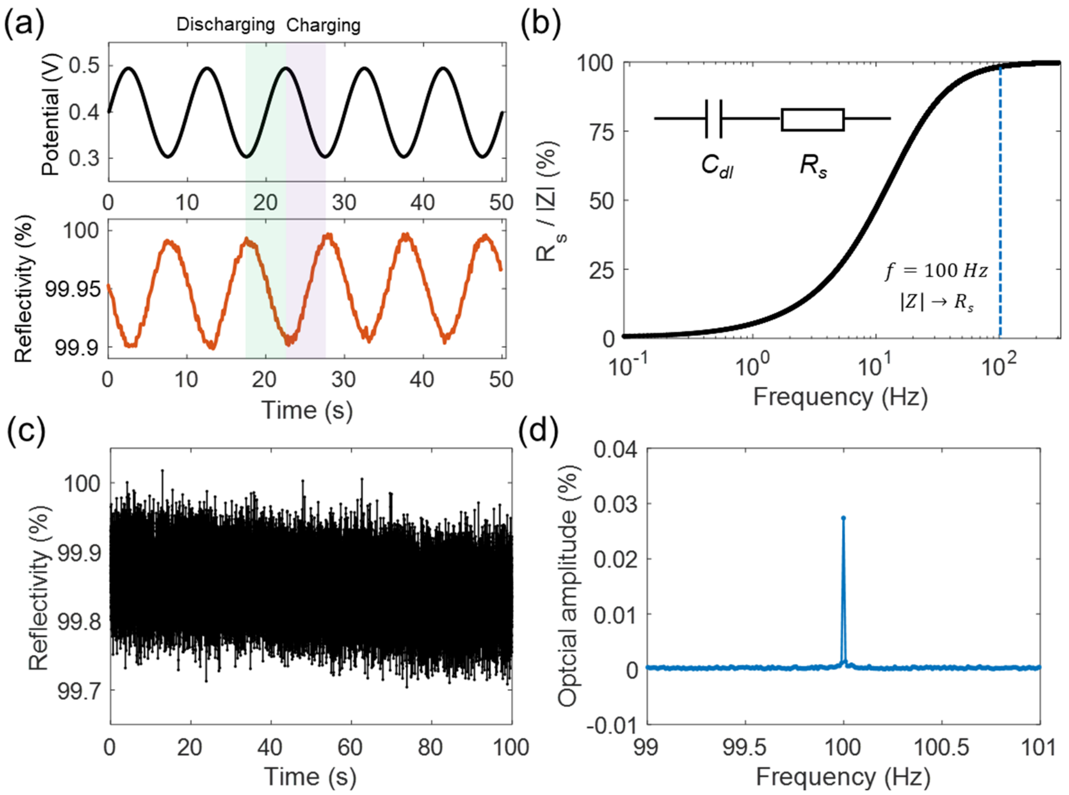

Supplementary Fig. 15. (a) Reflectivity of Au electrode as a function of sinusoidal potential modulation (frequency, 0.1 Hz; amplitude, 100 mV; offset, 400 mV). (b) At 100 Hz, the solution resistance is dominated among the total IZI. (c) Fluctuation of the reflectivity intensity for 100 Hz modulation and (d) optical amplitude extracted after Fourier transform.

Under this potential condition, the electrode mainly experienced the establishment of the electric double layer (EDL), which involved the migration of electrons alongside the conformational metamorphosis, spatial reorganization and electromigration of ions under the compelling influence of the electric field, resulting in the formation of a compact layer plus a diffuse layer [8]. The higher optical amplitude observed at the periphery at high frequency indicated an accelerated charging rate compared to the central area within the same timescales (Supplementary Fig. 16c). This accelerated charging also meant a rapid accumulation of electrons and ions, facilitating the formation of a distinct positive-negative charge-separated EDL structure, thereby resulting in a more pronounced optical response. As the reaction proceeded to lower frequencies (f =1/t), the charging at each specific location neared completion, leading to relatively uniform optical signals. The notable discrepancy in optical amplitude between the edge and central regions can be attributed to the variation in electric field intensity, notably more pronounced at the periphery. Further detailed insights into this phenomenon would be discussed in the Note 13.

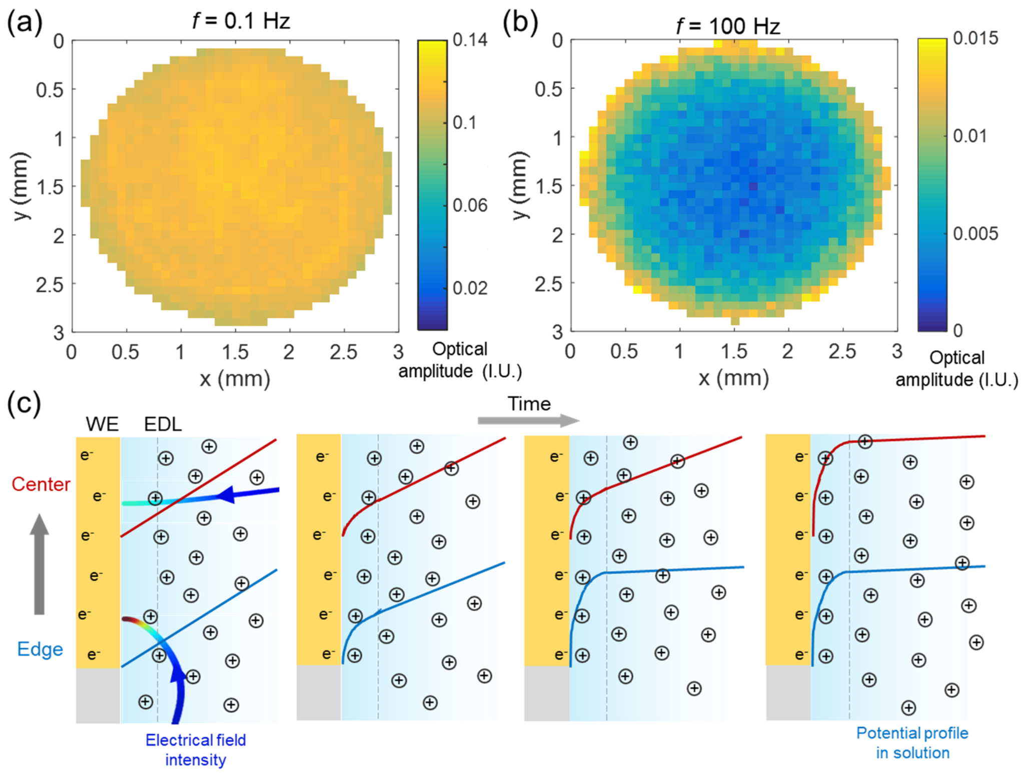

Supplementary Fig. 16. Planar distribution of optical amplitude over the electrode surface at (a) 0.1 Hz and (b) 100 Hz. (c) Scheme of the process of the electric double layer formation at both the edge and central regions, alongside the establishment of potential on the solution side.

Since directly measuring the optical current through a first-order derivative is challenging [6], a mathematical transformation is accordingly devised to obtain the impedance (based on voltage and current) from the optical amplitude (corresponding to charge quantity or the integration of current). Specifically, the amplitude (IZI) and phase (φ_Z) of the impedance can be written as:

$$|Z| = \left|\frac{V}{I}\right| = \left|\frac{V}{Q\cdot 2\pi f}\right| = \left|\frac{V}{\alpha\cdot opt\cdot 2\pi f}\right| \propto \left|\frac{1}{opt\cdot 2\pi f}\right| \quad (2)$$

$$\varphi_Z = \varphi_V - \varphi_I = \varphi_V - \varphi_Q - \frac{\pi}{2} \quad (3)$$

where f is the potential modulation frequency, V is the applied potential, α is the photoelectric conversion coefficient, Q is the charge transferred during the process and is considered to be proportional to the measured reflectivity amplitude *opt* [1,6]. As such, the measured optical amplitude was directly converted to the relative scale of overall impedance. The equivalent circuit depicted in Fig. 4a was employed to understand the charging process of Au electrode, which consisted of a double layer capacitor (C_{dl}) and a solution resistor (R_s):

$$Z = R_s + \frac{1}{j\omega C_{dl}} \quad (4)$$

This equivalent circuit is commonly adopted to explain the charging process of a non-Faradic reaction [5]. Notably, the solution resistance was found to dominate the overall impedance at 100 Hz according to equivalent circuit analysis, accounting for 98% of the total IZI (Supplementary Fig. 15b). On the other hand, the double layer capacitor was found to dominate the overall impedance at 0.1 Hz correspondingly.

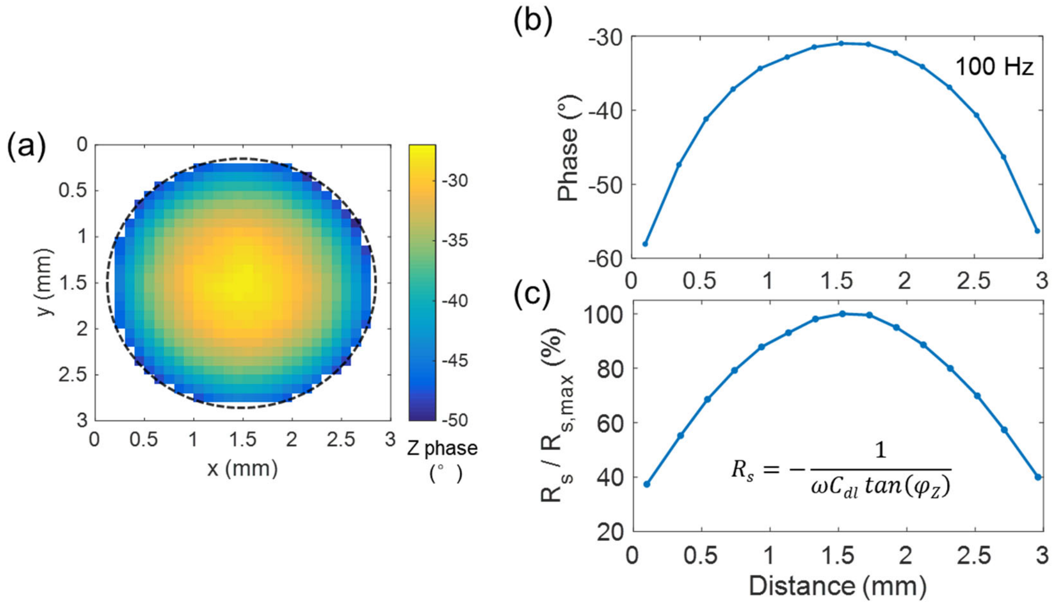

Supplementary Fig. 17. (a) Planar distribution of phase over the electrode surface. (b) The radial distribution of extracted phase value and (c) calculated normalized solution resistance.

Technically, we also extract the phase variation from the potential modulated optical intensity changes since as compared to the optical amplitude data, the phase measurements exhibit little inference from the short-term noise and thus superior signal-to-noise ratios (Supplementary Fig. 17a). Fundamentally, the phase is derived from the temporal delay between two signals, making it less affected by background intensity in comparison to amplitude [10] and less limited by the illumination conditions described in Note 10. Therefore, the phase shift at each site of the electrode surface in 100 Hz is extracted (Supplementary Fig. 17b) and mathematically converted to R_s accordingly:

$$tan(\varphi_Z) = -\frac{1}{\omega R_s C_{dl}},\ R_s = -\frac{1}{\omega C_{dl} \cdot tan\,(\varphi_Z)} \quad (5)$$

Since C_{dl} was found to remain unchanged, the distribution of R_s was depicted in Supplementary Fig. 17c. The extraction of the distribution of R_s using phase data at 100 Hz offered several advantages, including higher quality, stability and experimental convenience. Subsequently, this phase processing method was used to obtain the distribution of R_s in the following experiments.

12. R_s distribution on a GC electrode

The same experiment in sulfuric acid solution has also been conducted on a well-cleaned glassy carbon (GC) electrode, except the use of a 730 nm LED as the light source due to the overall suitable sensitivity and quantum efficiency at the wavelength. Consequently, as the potential was scanned positively from 0.05 V to 1.4 V, the average reflectivity exhibited a decline from its initial value of 100% to 99.2% (Supplementary Fig. 18a). By similarly measuring the spatial distribution of impedance during the charging process in high-frequency domain (and thus that of the solution resistance), we observed the same phenomenon in the GC electrode system (Supplementary Fig. 18b).

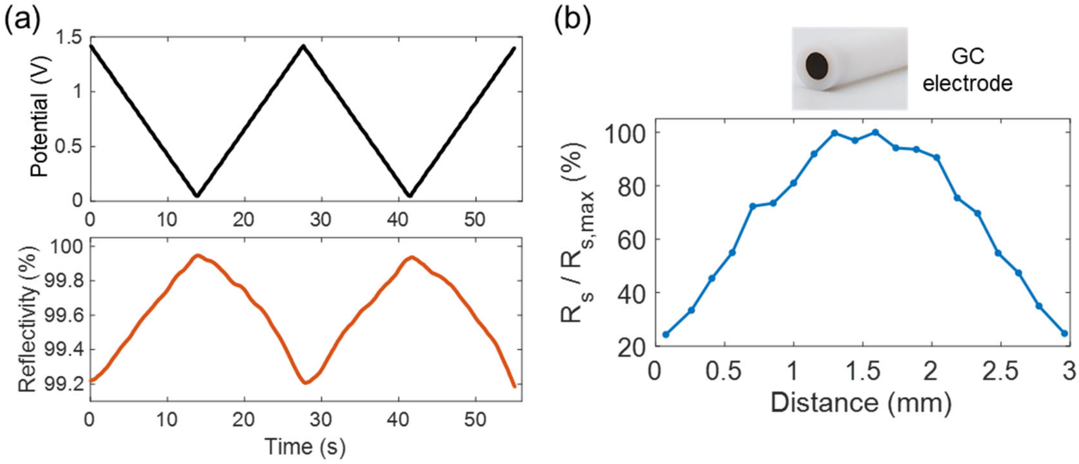

Supplementary Fig. 18. (a) Mean reflectivity of the overall GC electrode surface as a function of applied electrode potential (vs. Ag/AgCl), scan rate: 100 mV/s. (b) Radial distribution of normalized solution resistance over the GC electrode surface.

13. Theoretical modelling based on COMSOL

In the three-electrode system, the current passing between the WE and RE is extremely small, with the RE primarily tasked with setting the precise potential at the WE. It is WE and CE that form the circuit and current can flow. Thus, the electric field distribution we simulated was situated between WE and CE.

In the case of symmetrically positioned parallel-plate electrode of equal dimension, the electric field lines were evenly distributed between the electrodes, resulting in a linear variation of potential [11]. In contrast, in the traditional three-electrode setup, WE and CE were asymmetrically positioned, with the CE having a significantly larger electrode area than the WE. Consequently, the spatial distribution of the electric field

exhibited following characteristics: electric field lines were radially distributed around the near-electrode space, with the voltage drop primarily concentrated near the WE (Fig. 5a). At the same time, the electric field intensity at the electrode periphery was notably stronger than that in the central region (Supplementary Fig. 19a). The unique high electric field intensity at the electrode edge can be attributed to the significant bending and higher curvature of the electric field lines at this location, in contrast to the relatively straight and lower curvature of the lines in the central region. This disparity in curvature resulted in a weaker electric field intensity in the central region [12] (Supplementary Fig. 19b).

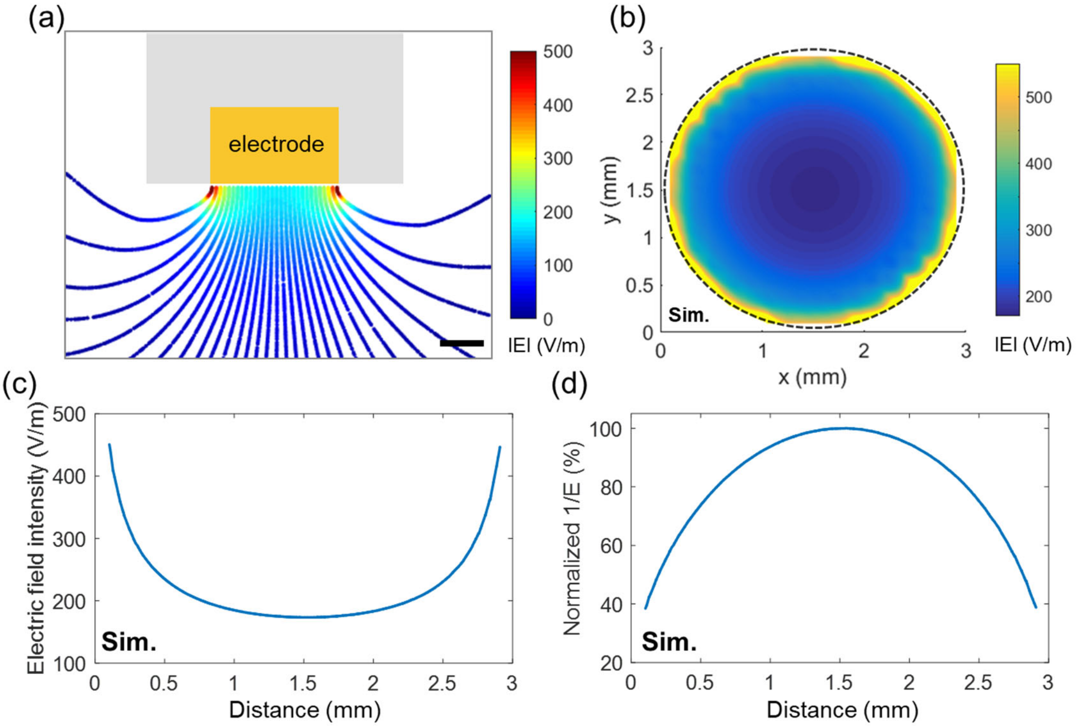

Supplementary Fig. 19. (a) The cross-section of electric field intensity near the working electrode, scale bar: 0.5 mm.(b) Planar distribution of electric field intensity at the working electrode. (c) Radial distribution curve of the electric field intensity and (d) the normalization of the reciprocal of electric field intensity.

Meanwhile, the ion mobility is governed by the intensity of electric field. A direct correlation existed between the electric field intensity (E) and the migration rate (μ) of ions ($\mu \propto E$), indicating that higher electric field intensity lead to faster ion migration, thereby reducing the time needed to establish the EDL. This relationship allowed us to link the solution resistance (R_s) and electric field intensity (E) through the time constant τ, which can be expressed as follows:

$$\tau = C_{dl}R_s \propto \frac{1}{\mu} \propto \frac{1}{E} \tag{6}$$

Consequently, increased electric field intensity resulted in reduced barriers for ion transport, lower solution resistance, and quicker establishment of the EDL ($R_s \propto 1/E$). By converting the electric field intensity results (Supplementary Fig. 19c) into a distribution of solution resistance, we found a strong agreement with our experimental findings (Supplementary Fig. 19d).

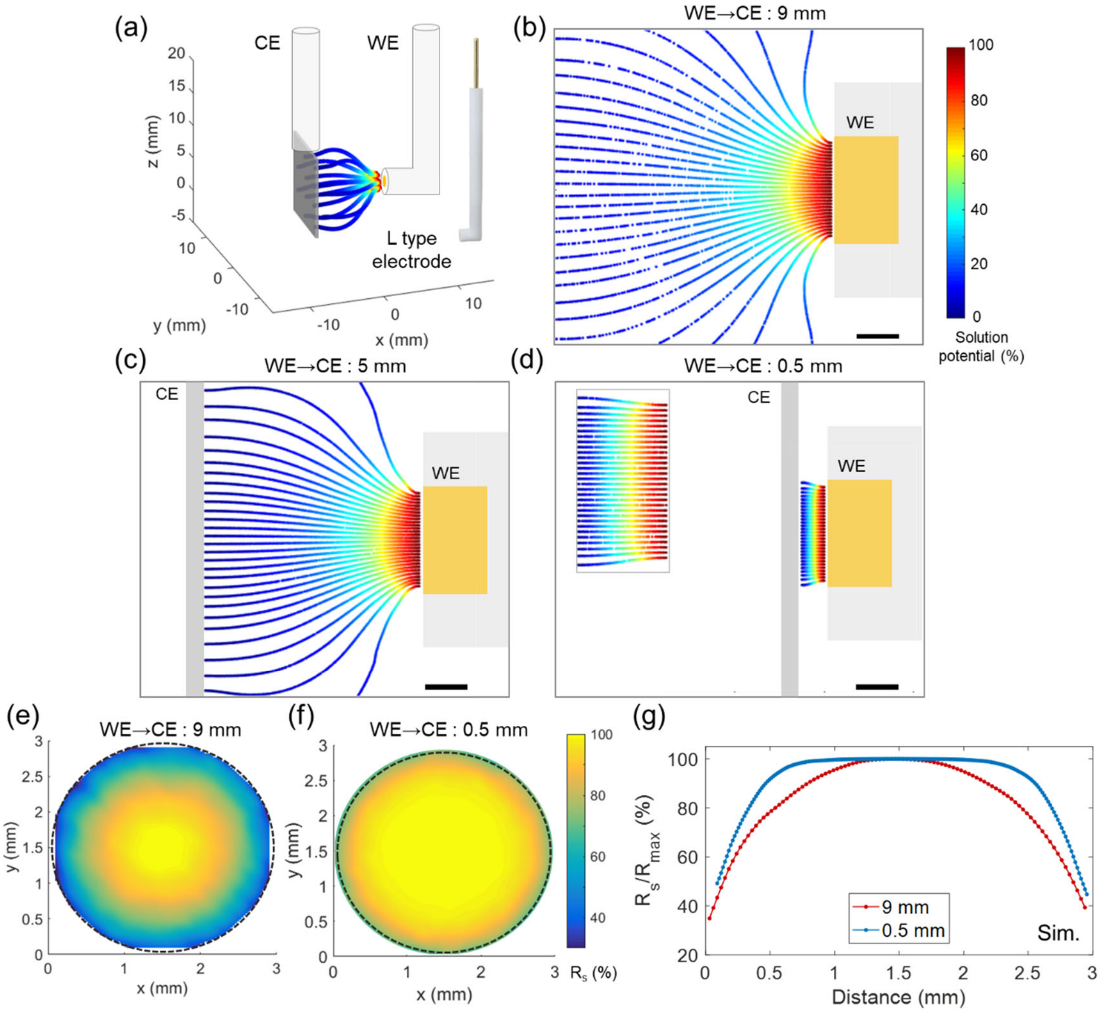

Supplementary Fig. 20. (a) COMSOL simulated 3D plot of the distribution of electric field line between the L-type working electrode and the counter electrode. (b) Cross-sectional images of electric field lines at WE distances of (b) 9 mm, (c) 5 mm and (d) 0.5 mm from CE, scale bar: 1 mm. Planar distributions of solution resistance at the electrodes at distances of (e) 9 mm and (f) 0.5 mm. (g) Radial distribution curves of solution resistance at distances of 9 mm and 0.5 mm accordingly.

Furthermore, we explored the impact of electrode placement on the distribution of electric field intensity and solution resistance. When positioning WE and CE in a 'face-to-face' configuration in COMSOL (usually utilizing L-shaped electrodes in the experiment), the same radial distribution of electric field lines was observed

(Supplementary Fig.20a). Reducing the distance between WE and CE from 9 mm to 0.5 mm (Supplementary Fig.20 b-d) resulted in a convergence of the electric field lines, with reduced curvature at the edges that resembled the parallel plate electrodes. The electric field lines were evenly distributed between the two electrodes, maintaining consistent lengths with only minor deviations in the edge position (Supplementary Fig.20d). The calculated R_s distributions also indicated that decreasing the distance between WE and CE led to a reduction in the disparity of solution resistance within the central region of the electrode (Supplementary Fig.20 e-g).

The simulation outcomes obtained from the 'face-to-face' configuration of WE and CE highlighted that the presence of heterogeneous solution resistance primarily stemmed from the mismatch in size between WE and CE, which was difficult to avoid experimentally. Because the area of CE was always deliberately designed to be as large as possible in order to reduce the current density, ensuring it remained unpolarized during measurements and did not interfere with the reaction of WE.

14. R_s distribution on the electrode passivated with an insulating boundary

In light of the discussion in the Note 13, our investigation revealed that the uneven distribution of the electric field across the electrode surface contributed to the variability in solution resistance, with the electric field intensity notably higher in the edge region than in the central region. To explore this phenomenon further, we deliberately introduced an insulating strip in the central area along the 6 mm diameter disc electrode using a black marker pen, creating a barrier approximately 0.8 mm wide (Supplementary Fig. 21a).

Subsequent to conducting an oEIS test on this ‘modified electrode’, the resulting distribution of solution resistance on the electrode surface was depicted in Supplementary Fig. 21b. Notably, the location previously designated as the center of the electrode (labelled as Center1) transitioned to the edge position following the introduction of the insulating interface. Conversely, a new central region, labelled as Center2, emerged at the midpoint of the divided semicircle. This observation suggested that edge regions were not confined solely to the outer periphery of the electrode but extended to the regions that interface with the insulating region. In these regions, the electric field lines were bent due to the presence of the insulating region, resulting in a slightly higher electric field intensity compared to regions located farther from the edges (Supplementary Fig. 21a&b).

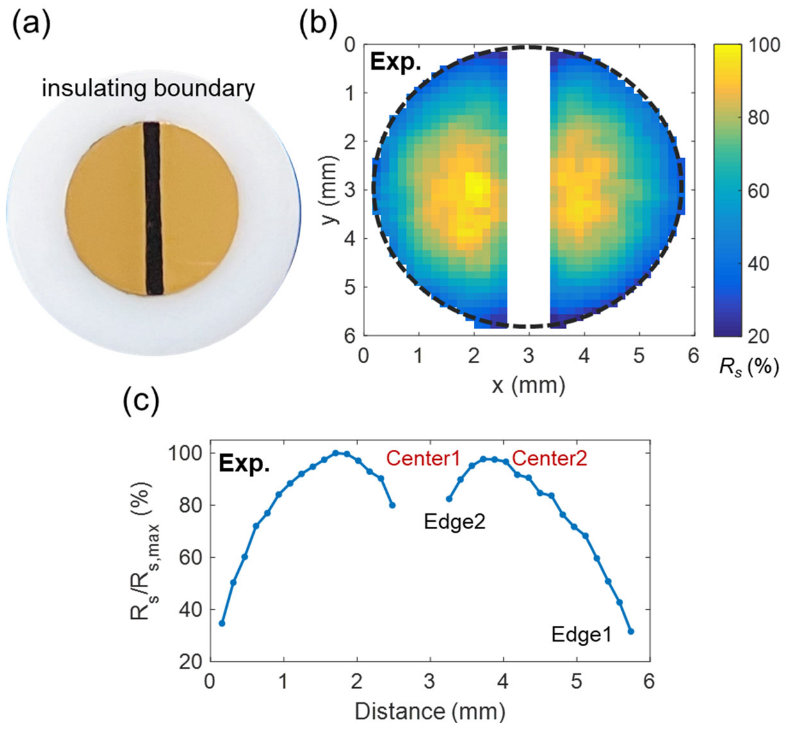

Supplementary Fig. 21. (a) Photograph of the marked 6 mm diameter gold electrode. (b) Corresponding mapping and (c) distribution along a horizontal diameter of the solution resistances with highlighted two separate centers and the new edges of the distribution.

It was noteworthy that the solution resistance at the newly defined edge position created by the insulating strip (labelled as Edge2) differed from that at the original edge position adjacent to the PTFE insulating layer (labeled as Edge1) (Supplementary Fig. 21c). This discrepancy may be attributed to the distinct locations of these two edges on the electrode: Edge1 resided at the periphery of the disc electrode, where the curvature of the electric field lines was more pronounced; whereas Edge2 was situated at the center of the electrode. Despite the presence of insulating strips, the curvature of the electric field lines at Edge2 remained comparatively slight, resulting in lower electric field intensity. Nevertheless, the curvature of the electric field lines at Edge2 surpassed that of the electric field lines at Center2, thereby yielding a higher electric field intensity in this region. These results are well consistent with those simulated in Supplementary Fig. 22.

On the other hand, as we established a correlation between solution resistance and electric field intensity, we assumed that the distribution of electric field lines between WE and CE would remain unaffected by solution diffusion and convection. Therefore, the variability in solution resistance could be equally observed within a flowing solution system (as discussed in Note 7).

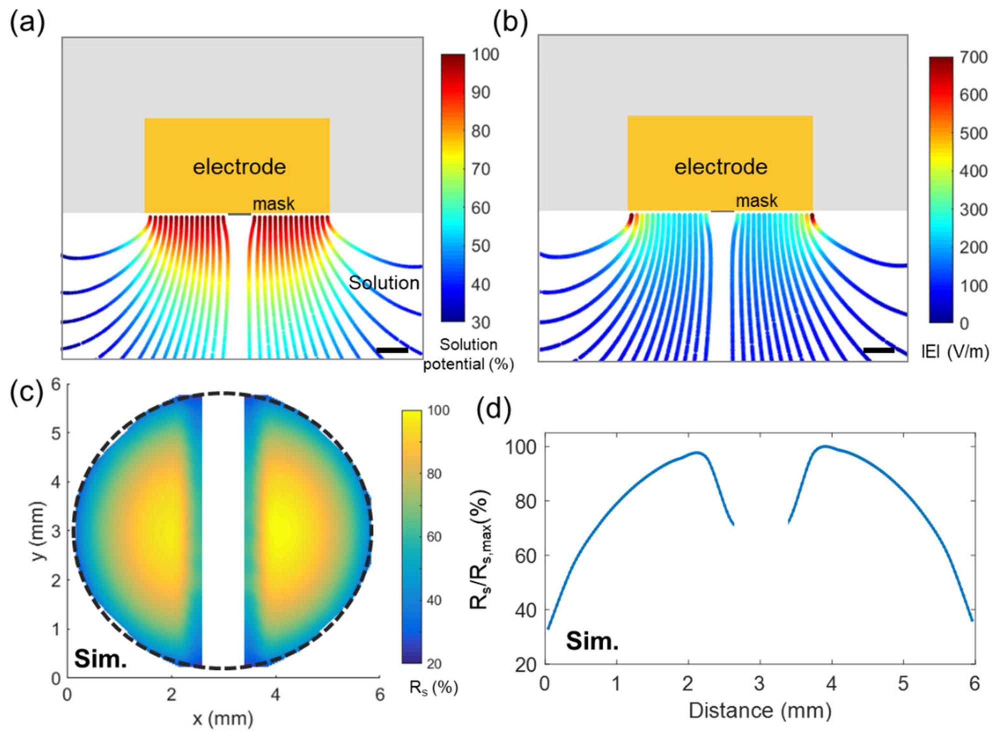

Supplementary Fig. 22. Simulated (a) cross-sectional images of solution potential and (b) electric field intensity, scale bar: 1 mm. (c) Solution resistance mapping and (d) radial distribution curve from simulated results.

15. *R_s* distributions on the retracted electrode

To further support the preceding discussion, we have devised a retracted electrode that effectively eliminates the temporal heterogeneity arising from varying solution resistance (R_s) values. The electrode is composed of a threaded copper rod, a 3 mm diameter gold electrode, and a polytetrafluoroethylene casing (as shown in Supplementary Fig. 23c). By manipulating the copper rod, the gold electrode can be concaved into the casing. In our experimental setup, we retract the gold surface by 2 mm inward from the leveled plane of the PTFE surface. This retraction significantly eliminates the disparity in peak potential between the edge and central regions, as illustrated in Fig. 6b.

Furthermore, the results are supported by the simulated outcomes of the solution potential distribution and electric field intensity distribution, demonstrating that the potential drop near the electrode surface is now completely confined within the created channel and that the electric field intensity on the electrode surface remains relatively consistent (Supplementary Fig. 23a&b). Consequently, the radical disparities in solution resistance observed in the oEIS experiment are nearly eliminated (Supplementary Fig. 20d).

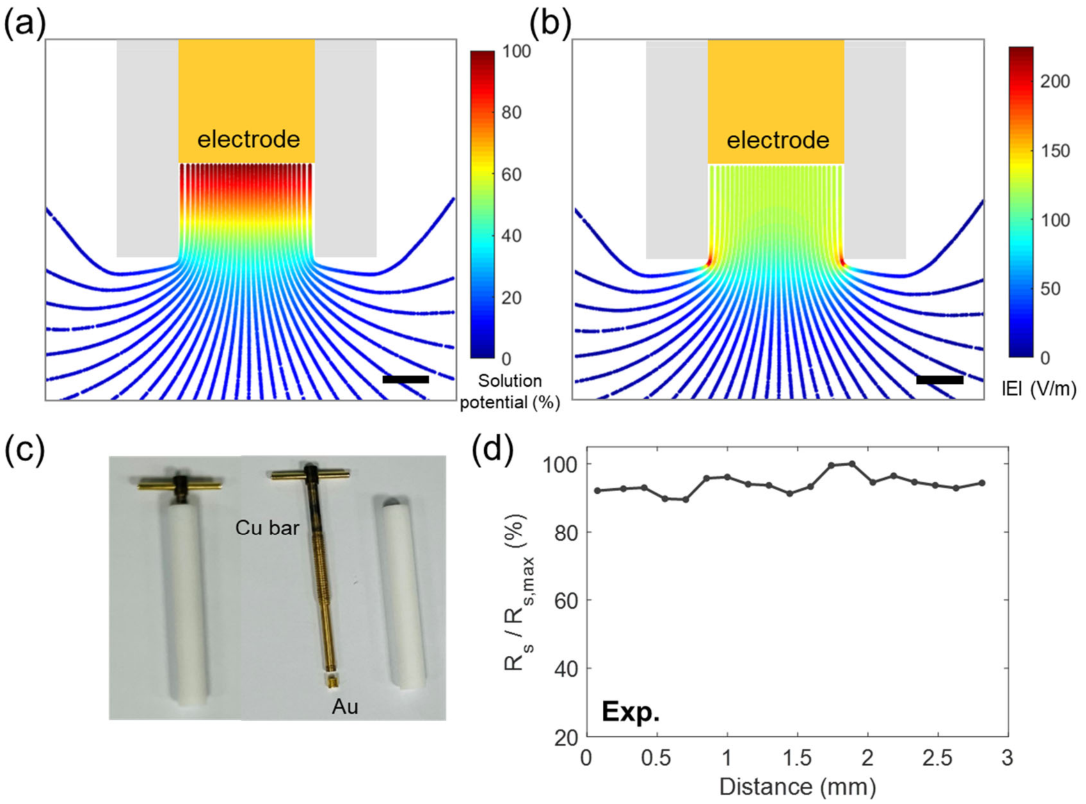

Supplementary Fig. 23. (a) COMSOL simulation of the near-electrode potential distribution and (b) electric field intensity distribution from the retracted electrode, scale bar: 0.5 mm. (c) The physical image of the retracted electrode. (d) The corresponding radial solution resistance distribution measured in oEIS experiment.

Supplementary References

[1]. Johnson, P. B.; Christy R. W., Optical Constants of the Noble Metals. *Phys. Rev. B* **6**, 4370-4379 (1972).

[2]. Noginov, M. A.; Zhu, G.; Gavrilenko, V. I., Sensitized nonlinear emission of gold nanoparticles. *Opt. Express* **15**, 15648-15655 (2007).

[3]. Shi, Y.; Feng, G.; Li, X.; Yang, X.; Ghanim, A. H.; Ruchhoeft, P.; Jackson, D.; Mubeen, S.; Shan, X., Electrochemical Impedance Imaging on Conductive Surfaces. *Anal. Chem.* **93**, 12320-12328 (2021).

[4]. Jiang, D. et al. Optical Imaging of Phase Transition and Li-Ion Diffusion Kinetics of Single $LiCoO_2$ Nanoparticles During Electrochemical Cycling. *J. Am. Chem. Soc.* **139**, 186-192 (2017).

[5]. Du, D., Wang, M., Qin, Y. & Lin, Y. One-step electrochemical deposition of Prussian Blue–multiwalled carbon nanotube nanocomposite thin-film: preparation, characterization and evaluation for H_2O_2 sensing. *J. Mater. Chem.* **20**, 1532-1537 (2010).

[6]. Niu, B. et al. Determining the depth of surface charging layer of single Prussian blue nanoparticles with pseudocapacitive behaviors. *Nature Commun.* **13**, 2316 (2022).

[7]. Tremiliosi-Filho, G., Dall'Antonia, L. H. & Jerkiewicz, G. Growth of surface oxides on gold electrodes under well-defined potential, time and temperature conditions. *J. Electroanal. Chem.* **578**, 1-8 (2005).

[8]. Bard, A. J.; Faulkner, L. R., Electrochemical Methods: Fundamentals and Applications. 2nd ed.; John Wiley & Sons.: 2002.

[9]. Liu, T., Li, M., Wang, Y., Fang, Y. & Wang, W. Electrochemical impedance spectroscopy of single Au nanorods. *Chem. Sci.* **9**, 4424-4429 (2018).

[10]. MacGriff, C. et al. Charge-Based Detection of Small Molecules by Plasmonic-Based Electrochemical Impedance Microscopy. *Anal. Chem.* **85**, 6682-6687 (2013).

[11]. Yang, G., Deng, D., Zhang, Y., Zhu, Q. & Cai, J. Numerical Optimization of Electrodeposition Thickness Uniformity with Respect to the Layout of Anode and Cathode. *Electrocatalysis* **12**, 478-488 (2021).

[12]. Purcell, E. M. and D. J. Morin. Electricity and Magnetism. Cambridge, Cambridge University Press: 2013.
